# Correction: MKP2 inhibits TGF-β1-induced epithelial-to-mesenchymal transition in renal tubular epithelial cells through a JNK-dependent pathway

**DOI:** 10.1042/CS-2018-0602_COR

**Published:** 2023-10-20

**Keywords:** epithelial-to-mesenchymal transition, mitogen-activated protein kinases, renal fibrosis, transforming growth factors

The authors of the original article, “MKP2 inhibits TGF-β1-induced epithelial-to-mesenchymal transition in renal tubular epithelial cells through a JNK-dependent pathway” (doi: 10.1042/CS20180602), would like to correct their paper.

The authors have been made aware of an error regarding the two images of control group in Figure 2D, and would like to apologise to readers for this error which does not affect the overall conclusion of the article.

**Figure 2:**
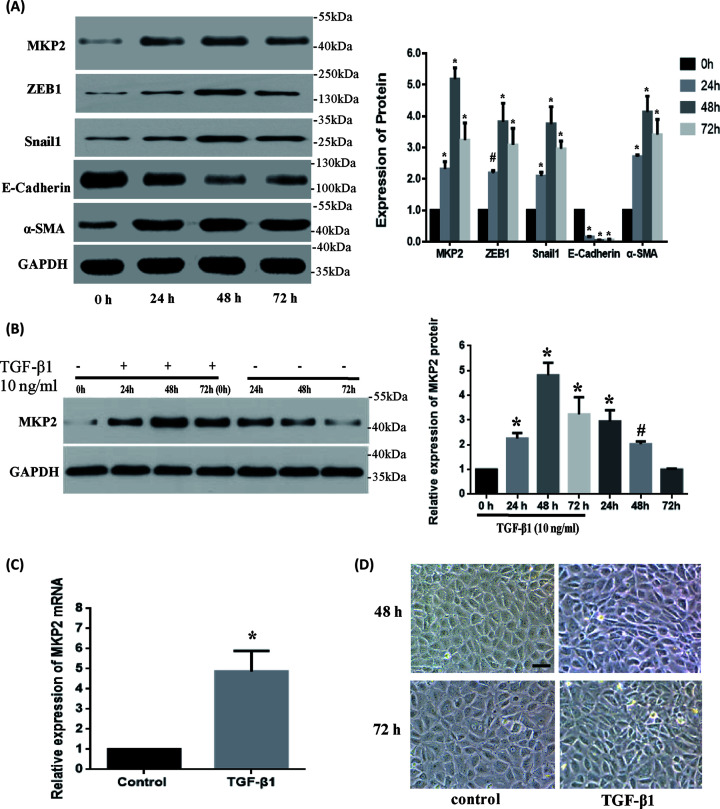
MKP2 is up-regulated in TGF-β1-induced EMT

The corrected Figure 2 is provided here:

